# Grand challenges in sex differences in immunology: problems the field must solve before biological sex can guide clinical practice

**DOI:** 10.3389/fimmu.2026.1965393

**Published:** 2026-08-26

**Authors:** Katie L. Flanagan, Susan Kovats

**Affiliations:** 1 Sydney Infectious Diseases Institute, Faculty of Health and Medicine, University of Sydney, Sydney, NSW, Australia; 2 Centre for Infectious Diseases and Microbiology, Westmead Hospital, Sydney, NSW, Australia; 3 Cancer Ageing and Vaccines (CAVA) Laboratory, School of Health and Biomedical Sciences, Royal Melbourne Institute of Technology (RMIT) University, Melbourne, VIC, Australia; 4 School of Medicine, College of Health and Medicine, University of Tasmania, Hobart, TAS, Australia; 5 Arthritis & Clinical Immunology Program, Oklahoma Medical Research Foundation, Oklahoma City, OK, United States

**Keywords:** epigenetics, life course, microbiome, sex chromosomes, sex steroids

## Abstract

Male and female immune systems differ in composition and function across the entire life course, shaping susceptibility to infection, autoimmunity, allergy, neurodegeneration, cancer, and responses to vaccines and immunotherapies. Despite decades of observational evidence, mechanistic clarity on why these differences arise - and how to exploit them therapeutically - remains limited. This paper outlines six grand challenges that, if addressed, would move sex-differential immunology from description toward clinical application: (1) rigorous identification of the sex differences in immune cell function across the lifespan during health and disease; (2) defining the functional roles of X- and Y-linked immune genes and the impact of X chromosome inactivation escape; (3) mapping the role of sex steroids including their contribution to sex-specific epigenetic regulation; (4) clarifying the immunological impact of the bidirectional sex–microbiota–immunity axis; (5) ensuring that experimental models and study designs make sex a variable rather than a confounder; and (6) translating mechanistic insights into sex-tailored disease prevention and treatment. Progress on these fronts could improve outcomes for both sexes across infectious disease, autoimmunity, allergy, neurodegeneration, cancer, and vaccinology.

## Introduction

Sex differences in immunity are documented from birth through old age, encompassing nearly every immune cell subset and function (1, 2). Women generally mount stronger innate and antibody responses than men, particularly during reproductive years, while immune shifts toward a more tolerogenic state during pregnancy (3, 4). Twin and population studies indicate that most of this variation is non-heritable, implicating environmental and hormonal exposure rather than germline genetics (5). Advances in multi-omic profiling, single-cell phenotyping, cell type-specific sex steroid receptor knockouts, four-core-genotype animal models, and human cohorts undergoing hormone-replacement, hormone-blockade, or gender-affirming hormone therapy now allow these differences to be explained, not just documented. This paper highlights six grand challenges that, if answered, would pave the way for immunological sex differences to inform clinical care.

## Challenge 1: getting the details right: precise identification of the sex differences in immune cell functional dynamics across the lifespan in health and disease

Sex differences in immune cell differentiation, numbers, or functional responses may be observed during homeostasis or manifest in the context of a specific disease state or challenge with pathogens or vaccines [reviewed in (6–12)]. The net immune response is driven by the collaboration between innate and adaptive immune cells. Each cell type, or pathways within them, may be differentially regulated by sex-related factors including sex steroids and sex chromosomes acting simultaneously, making their individual contributions difficult to disentangle. Nevertheless, clear pathways have been described such as estrogen acting via cell-intrinsic estrogen receptor alpha (ERα) to promote elevated type I IFN production by dendritic cells in females (13), and the X chromosome linked epigenetic regulator UTX, which escapes X inactivation and governs NK cells (14).

Estrogens tend to be pro-inflammatory at low concentrations and anti-inflammatory at high concentrations, while androgens are largely immunosuppressive, though both hormone classes have context-dependent and sometimes opposing effects depending on cell type and tissue (15). Sex disparate regulation of immune cells may occur in a specific tissue landscape or arise from indirect effects of sex steroids. A female sex bias may be explained by the presence of estrogens or the absence of androgens. Thus, the answers are in the details, and careful observation and experimentation are essential to map out sex-dependent regulation of immunity and avoid overly broad generalization.

Observed sex differences in immunity vary with age across the lifespan. Some differences are present from birth and persist throughout life, while others, such as greater B cell activity in women, emerge specifically at puberty, implicating a hormonal trigger (16). Other differences have been linked to the stage of the menstrual or estrous cycle (17). Sex differences do not disappear after menopause: post-menopausal women continue to show a distinct leukocyte composition compared with age-matched men, and life-stage-specific patterns of low-grade chronic inflammation (‘inflammaging’) also differ by sex (18–20). Cross-sectional studies of healthy adults reporting sex differences in immune cell frequencies are often contradictory and longitudinal studies would be more helpful (21, 22). Studies of people undergoing gender-affirming or menopausal hormone therapy demonstrate how shifting hormone exposure reshapes immune cell composition and function within the same individual over time, helping separate hormonal from chromosomal contributions prospectively (23–25).

## Challenge 2: elucidating the impact of sex-linked immune genes and incomplete X chromosome inactivation

The X chromosome carries many key immune-related genes, and while one X chromosome in each cell of a female is normally silenced through X chromosome inactivation, some genes escape this silencing and are expressed biallelically in women (9, 26). The molecular architecture of the inactive X, including which genes it selectively protects from silencing, is increasingly well characterized in mouse and human cells (27, 28). Escape from inactivation is a leading candidate mechanism for female-biased autoimmunity: X-linked innate sensors such as *TLR7* and adaptor genes such as *CD40L* are overexpressed in immune cells from women with lupus (29, 30). The X chromosome also carries many microRNAs, several of which regulate dendritic cell survival, T cell signaling, and granulocyte development, with sex-differential expression patterns relevant to autoimmune disease and cancers such as melanoma (31–33). The Y chromosome has a smaller but non-trivial role: age-related mosaic loss of Y is associated with declining immune function, including reduced expression of leukocyte CD99, and worse infection and cancer outcomes in men (34, 35). A more complete characterization of the clinical implications of sex chromosome-linked immune gene expression could clarify sex differences across multiple disease pathologies and reveal new therapeutic targets.

## Challenge 3: mapping the roles of sex steroids, including epigenetic and transcriptional regulation by sex steroid receptors

Ligand-dependent nuclear receptors for estrogens (ER) and androgens (AR) mediate chromatin accessibility and gene expression through recruitment of histone-modifying coregulators and fate-determining transcription factors (36, 37). While many immune cells express ER and/or AR to varying degrees, expression may change with cellular activation, and the field is only beginning to elucidate ER- or AR-mediated regulation of the epigenome or transcriptome in specific cell types [reviewed in (11)]. Furthermore, hematopoietic stem and progenitor cells (HSPCs) also express ER and AR (38), raising the possibility that HSPC exposure to sex hormones creates durable epigenetic changes (DNA or histone modification) that modulate immune cell differentiation that persists in mature immune cells, including short-lived myeloid cells (39). Mechanisms will be informed by determination of whether a particular cellular response requires constant exposure to sex steroid levels (extrinsic effect) or is governed epigenetically via a historical sex steroid exposure (intrinsic effect) (40, 41).

ER and AR activity also may mediate the extensive sex- and cell-type-specific patterns of DNA methylation that have been identified in genome-wide studies of human blood. The ongoing efforts to define epigenetic sexual dimorphism will increase our understanding of the differences in disease susceptibility between men and women (42–44). Another area of active investigation seeks to understand how sex steroid receptors regulate specific cellular pathways. For example, both ER and AR signaling regulate autophagy gene transcription directly, with the direction of the effect dependent on cell type, receptor subtype, and physiological state (45, 46). ER activity also regulates innate immune signaling via collaboration with NF-κB and promoting transcription of genes within the TLR-IFN axis (47).

## Challenge 4: the sex–microbiota–immunity axis

Gut microbial composition differs between males and females from birth, a phenomenon sometimes termed the ‘microgenderome’ or ‘microsexome’ (48, 49). Sex differences in microbial diversity are present in preterm and term infants and diverge further at puberty, implicating sex steroids as a driver (50, 51). Circulating sex steroid levels correlate with gut microbial diversity and composition in adults, supporting a bidirectional axis in which hormones shape the microbiota and microbial metabolism in turn alters hormone bioavailability (52, 53). This bidirectionality is visible across major hormonal transitions: the gut microbiome shifts substantially during pregnancy, and post-menopausal women develop a microbiota that more closely resembles that of men than of pre-menopausal women (54, 55). The functional consequences of this axis for immunity are still unclear, but the microbiota is implicated in shaping immune development and modulating responses to cancer immunotherapy, offering a plausible explanation for sex differences in treatment outcome (56–58).

## Challenge 5: fixing how sex is studied

A persistent challenge is that sex is still inconsistently reported or treated as a statistical nuisance rather than a biological variable, in both preclinical and clinical research (8, 12). Furthermore, the predominance of cross-sectional single-timepoint studies is poorly suited to capturing sex differences that emerge dynamically, and longitudinal designs spanning puberty, pregnancy, and menopause remain rare (5, 59). Correlating immune profiling with sex steroid levels in relation to cycling, age, and reproductive organ status would help resolve current inconsistencies. Treatment with sex steroids or selective ER or AR modulators *in vitro* is an attractive option for human cells, but requires careful titration of physiological levels in hormone-depleted culture medium (60). Pregnant individuals remain underrepresented in immunological research despite undergoing some of the most profound immune remodeling of the life course, leaving major gaps in understanding pregnancy-specific, sex-differential immunity (61, 62). Natural experiments - hormone blockade for cancer, hormone replacement for menopause or andropause, or gender-affirming hormone therapy - allow immune parameters to be tracked within the same individual as hormone exposure changes (63). Addressing these challenges requires sex-disaggregated reporting, deliberate inclusion of women and pregnant individuals in trials, and study designs that treat sex as an effect modifier to test, not a parameter to adjust away.

Murine models remain powerful for dissecting how sex steroid receptors and sex chromosomes shape the epigenome, transcriptome, and proteome, underlying sex differences in cellular composition and function of the immune system. Complementary *in vivo* approaches such as gonadectomy, sex steroid administration, cell type specific *Esr1*, *Esr2*, and *Ar* knockout mice, four-core genotype mice, humanized mice, and mixed bone marrow chimeras can help disentangle the complex sex-dependent mechanisms that govern immune cells.

Distinct immune cell types are likely regulated differently in distinct life stage, activation status, disease state, species, hence the roles of sex steroids or sex chromosomes in immune responses may vary accordingly. The field needs both hypothesis-driven animal or cellular models targeting specific pathways, and comprehensive immune profiling to generate new hypotheses and interpret human variation.

## Challenge 6: from mechanism to sex-tailored medicine

These mechanistic gaps translate into a practical one: clinicians have almost no tools or guidelines to tailor prevention or treatment by sex, despite clear evidence that vaccine response, autoimmune disease risk, and cancer therapy outcomes differ by sex (8, 64, 65). Women’s stronger vaccine antibody responses come with a higher rate of adverse events, a pattern documented across the life course prompting proposals for sex-adjusted dosing that remain largely untested (64). In cancer, sex differences in autophagy and the gut microbiome both plausibly shape responses to immunotherapy, and the current therapeutic use of androgen-receptor-targeted pathways that promote CD8+ T cell responses in prostate cancer illustrate that sex steroid biology can be directly exploited once the relevant mechanism is understood (57, 66–69). Closing this gap will require systems biology studies integrating hormonal, genetic, epigenetic, and microbial data within the same individuals over time, to define sex-specific biomarkers and thresholds for intervention with confidence.

## Conclusion

Sex differences in immunity are neither a footnote nor a confound — they are a fundamental axis of human immune variation with direct consequences for infectious disease, autoimmunity, allergy, neurodegeneration, metabolic dysregulation, cancer, and vaccination. The grand challenges outlined here are interconnected and mutually reinforcing. Meeting them will require sustained investment in longitudinal human cohorts, better animal and cellular models, and a research culture that treats biological sex as a primary variable rather than an afterthought. The prize is substantial: immunological care that is genuinely optimized for everyone, not calibrated by default to one sex and extrapolated to the other.

## References

[1] Klein SL Flanagan KL . Sex differences in immune responses. Nat Rev Immunol. (2016) 16:626–38. doi: 10.1038/nri.2016.90 27546235

[2] Dunn SE Perry WA Klein SL . Mechanisms and consequences of sex differences in immune responses. Nat Rev Nephrol. (2024) 20:37–55. doi: 10.1038/s41581-023-00787-w 37993681

[3] Hoffmann JP Liu JA Seddu K Klein SL . Sex hormone signaling and regulation of immune function. Immunity. (2023) 56:2472–91. doi: 10.1016/j.immuni.2023.10.008 37967530

[4] Creisher PS Klein SL . Pathogenesis of viral infections during pregnancy. Clin Microbiol Rev. (2024) 37:e0007323. doi: 10.1128/cmr.00073-23 38421182 PMC11237665

[5] Brodin P Jojic V Gao T Bhattacharya S Angel CJ Furman D . Variation in the human immune system is largely driven by non-heritable influences. Cell. (2015) 160:37–47. doi: 10.1016/j.cell.2014.12.020 25594173 PMC4302727

[6] McKernan KE Pilier EH Newcomb DC . Sex differences in asthma pathogenesis. Immunol Rev. (2026) 337:e70100. doi: 10.1111/imr.70100 41508394 PMC12783869

[7] Anesi N Miquel CH Laffont S Guery JC . The influence of sex hormones and X chromosome in immune responses. Curr Top Microbiol Immunol. (2023) 441:21–59. doi: 10.1007/978-3-031-35139-6_2 37695424

[8] Wilkinson NM Chen HC Lechner MG Su MA . Sex differences in immunity. Annu Rev Immunol. (2022) 40:75–94. doi: 10.1146/annurev-immunol-101320-125133 34985929 PMC9805670

[9] Forsyth KS Jiwrajka N Lovell CD Toothacre NE Anguera MC . The connexion between sex and immune responses. Nat Rev Immunol. (2024) 24:487–502. doi: 10.1038/s41577-024-00996-9 38383754 PMC11216897

[10] Voskuhl R Klein S . Sex is a biological variable - in the brain too. Nature. (2019) 568:171. doi: 10.1038/d41586-019-01141-6 30967673

[11] Miller RAJ Williams AP Kovats S . Sex chromosome complement and sex steroid signaling underlie sex differences in immunity to respiratory virus infection. Front Pharmacol. (2023) 14:1150282. doi: 10.3389/fphar.2023.1150282 37063266 PMC10097973

[12] Flanagan KL Klein SL . Knowledge gaps and research priorities to understand sex differences in immunity. PloS Biol. (2026) 24:e3003578. doi: 10.1371/journal.pbio.3003578 41628075 PMC12863523

[13] Laffont S Seillet C Guery JC . Estrogen receptor-dependent regulation of dendritic cell development and function. Front Immunol. (2017) 8:108. doi: 10.3389/fimmu.2017.00108 28239379 PMC5300975

[14] Cheng MI Li JH Riggan L Chen B Tafti RY Chin S . The X-linked epigenetic regulator UTX controls NK cell-intrinsic sex differences. Nat Immunol. (2023) 24:780–91. doi: 10.1038/s41590-023-01463-8 36928413 PMC12882737

[15] Quatrini L Ricci B Ciancaglini C Tumino N Moretta L . Regulation of the immune system development by glucocorticoids and sex hormones. Front Immunol. (2021) 12:672853. doi: 10.3389/fimmu.2021.672853 34248954 PMC8260976

[16] Fan H Dong G Zhao G Liu F Yao G Zhu Y . Gender differences of B cell signature in healthy subjects underlie disparities in incidence and course of SLE related to estrogen. J Immunol Res. (2014) 2014:814598. doi: 10.1155/2014/814598 24741625 PMC3987971

[17] Gockel CM Bao S Holland MK Beagley KW . Influence of the murine oestrous cycle on the induction of mucosal immunity. Am J Reprod Immunol. (2003) 50:369–79. doi: 10.1034/j.1600-0897.2003.00097.x 14750696

[18] Chen Y Zhang Y Zhao G Chen C Yang P Ye S . Difference in leukocyte composition between women before and after menopausal age, and distinct sexual dimorphism. PloS One. (2016) 11:e0162953. doi: 10.1371/journal.pone.0162953 27657912 PMC5033487

[19] Olivieri F Marchegiani F Matacchione G Giuliani A Ramini D Fazioli F . Sex/gender-related differences in inflammaging. Mech Ageing Dev. (2023) 211:111792. doi: 10.1016/j.mad.2023.111792 36806605

[20] Kang S Ko EY Andrews AE Shin JE Nance KJ Barman PK . Microglia undergo sex-dimorphic transcriptional and metabolic rewiring during aging. J Neuroinflamm. (2024) 21:150. doi: 10.1186/s12974-024-03130-7 38840206 PMC11155174

[21] Afshan G Afzal N Qureshi S . CD4+CD25(hi) regulatory T cells in healthy males and females mediate gender difference in the prevalence of autoimmune diseases. Clin Lab. (2012) 58:567–71. 22783590

[22] Melzer S Zachariae S Bocsi J Engel C Loffler M Tarnok A . Reference intervals for leukocyte subsets in adults: Results from a population-based study using 10-color flow cytometry. Cytometry B Clin Cytom. (2015) 88:270–81. doi: 10.1002/cyto.b.21234 25704947

[23] Lakshmikanth T Consiglio C Sardh F Forlin R Wang J Tan Z . Immune system adaptation during gender-affirming testosterone treatment. Nature. (2024) 633:155–64. doi: 10.1016/j.jri.2023.104065 39232147 PMC11374716

[24] De Maeyer RPH Sikora J Bracken OV Shih B Lloyd AF Peckham H . Age-associated inflammatory monocytes are increased in menopausal females and reversed by hormone replacement therapy. Aging Cell. (2025) 24:e70249. doi: 10.1101/2025.03.28.645904 41064932 PMC12611317

[25] Grunhagel B Borggrewe M Hagen SH Ziegler SM Henseling F Glau L . Reduction of IFN-I responses by plasmacytoid dendritic cells in a longitudinal trans men cohort. iScience. (2023) 26:108209. doi: 10.1016/j.isci.2023.108209 37953956 PMC10637924

[26] Fish EN . The X-files in immunity: sex-based differences predispose immune responses. Nat Rev Immunol. (2008) 8:737–44. doi: 10.1038/nri2394 18728636 PMC7097214

[27] San Roman AK Godfrey AK Skaletsky H Bellott DW Groff AF Harris HL . The human inactive X chromosome modulates expression of the active X chromosome. Cell Genom. (2023) 3:100259. doi: 10.1016/j.xgen.2023.100259 36819663 PMC9932992

[28] Forsyth KS Toothacre NE Jiwrajka N Driscoll AM Shallberg LA Cunningham-Rundles C . Maintenance of X chromosome inactivation after T cell activation requires NF-kappaB signaling. Sci Immunol. (2024) 9:eado0398. doi: 10.1126/sciimmunol.ado0398 39365876 PMC12088372

[29] Hewagama A Gorelik G Patel D Liyanarachchi P McCune WJ Somers E . Overexpression of X-linked genes in T cells from women with lupus. J Autoimmun. (2013) 41:60–71. doi: 10.1016/j.jaut.2012.12.006 23434382 PMC3622754

[30] Souyris M Cenac C Azar P Daviaud D Canivet A Grunenwald S . TLR7 escapes X chromosome inactivation in immune cells. Sci Immunol. (2018) 3. doi: 10.1126/sciimmunol.aap8855 29374079

[31] Grigoryev YA Kurian SM Hart T Nakorchevsky AA Chen C Campbell D . MicroRNA regulation of molecular networks mapped by global microRNA, mRNA, and protein expression in activated T lymphocytes. J Immunol. (2011) 187:2233–43. doi: 10.4049/jimmunol.1101233 21788445 PMC3159804

[32] Fattore L Costantini S Malpicci D Ruggiero CF Ascierto PA Croce CM . MicroRNAs in melanoma development and resistance to target therapy. Oncotarget. (2017) 8:22262–78. doi: 10.18632/oncotarget.14763 28118616 PMC5400662

[33] Dai R Ahmed SA . Sexual dimorphism of miRNA expression: a new perspective in understanding the sex bias of autoimmune diseases. Ther Clin Risk Manag. (2014) 10:151–63. doi: 10.2147/tcrm.s33517 24623979 PMC3949753

[34] Wang Y Sano S . Why Y matters? The implication of loss of Y chromosome in blood and cancer. Cancer Sci. (2024) 115:706–14. doi: 10.1111/cas.16072 38258457 PMC10921008

[35] Mattisson J Danielsson M Hammond M Davies H Gallant CJ Nordlund J . Leukocytes with chromosome Y loss have reduced abundance of the cell surface immunoprotein CD99. Sci Rep. (2021) 11:15160. doi: 10.1038/s41598-021-94588-5 34312421 PMC8313698

[36] Pihlajamaa P Sahu B Janne OA . Determinants of receptor- and tissue-specific actions in androgen signaling. Endocr Rev. (2015) 36:357–84. doi: 10.1210/er.2015-1034 26052734

[37] Mann M Cortez V Vadlamudi RK . Epigenetics of estrogen receptor signaling: role in hormonal cancer progression and therapy. Cancers (Basel). (2011) 3:1691–707. doi: 10.3390/cancers3021691 21814622 PMC3147309

[38] Nakada D Oguro H Levi BP Ryan N Kitano A Saitoh Y . Oestrogen increases haematopoietic stem-cell self-renewal in females and during pregnancy. Nature. (2014) 505:555–8. doi: 10.1038/nature12932 24451543 PMC4015622

[39] Singer K Maley N Mergian T DelProposto J Cho KW Zamarron BF . Differences in hematopoietic stem cells contribute to sexually dimorphic inflammatory responses to high fat diet-induced obesity. J Biol Chem. (2015) 290:13250–62. doi: 10.1074/jbc.m114.634568 25869128 PMC4505578

[40] Williams AP Turner S Chlebicz M Carter H Miller RAJ Allushi B . Cutting edge: Extrinsic and intrinsic sex effects differentially regulate pulmonary ILC2 numbers, phenotype, and function. J Immunol. (2025) 214:1891–7. doi: 10.1093/jimmun/vkaf114 40489769 PMC12394977

[41] Arnold AP . The organizational-activational hypothesis as the foundation for a unified theory of sexual differentiation of all mammalian tissues. Horm Behav. (2009) 55:570–81. doi: 10.1016/j.yhbeh.2009.03.011 19446073 PMC3671905

[42] Mamrut S Avidan N Staun-Ram E Ginzburg E Truffault F Berrih-Aknin S . Integrative analysis of methylome and transcriptome in human blood identifies extensive sex- and immune cell-specific differentially methylated regions. Epigenetics. (2015) 10:943–57. doi: 10.1080/15592294.2015.1084462 26291385 PMC4844205

[43] Bhattacharya S Sadhukhan D Saraswathy R . Role of sex in immune response and epigenetic mechanisms. Epigenet Chromatin. (2024) 17:1. doi: 10.1186/s13072-024-00525-x 38247002 PMC10802034

[44] Migliore L Nicoli V Stoccoro A . Gender specific differences in disease susceptibility: The role of epigenetics. Biomedicines. (2021) 9. doi: 10.3390/biomedicines9060652 34200989 PMC8228628

[45] Xiang J Liu X Ren J Chen K Wang HL Miao YY . How does estrogen work on autophagy? Autophagy. (2019) 15:197–211. doi: 10.1080/15548627.2018.1520549 30208759 PMC6333457

[46] Shang D Wang L Klionsky DJ Cheng H Zhou R . Sex differences in autophagy-mediated diseases: toward precision medicine. Autophagy. (2021) 17:1065–76. doi: 10.1080/15548627.2020.1752511 32264724 PMC8143224

[47] Kovats S . Estrogen receptors regulate innate immune cells and signaling pathways. Cell Immunol. (2015) 294:63–9. doi: 10.1016/j.cellimm.2015.01.018 25682174 PMC4380804

[48] Vemuri R Sylvia KE Klein SL Forster SC Plebanski M Eri R . The microgenderome revealed: sex differences in bidirectional interactions between the microbiota, hormones, immunity and disease susceptibility. Semin Immunopathol. (2019) 41:265–75. doi: 10.1007/s00281-018-0716-7 30298433 PMC6500089

[49] Mulak A Larauche M Tache Y . Sexual dimorphism in the gut microbiome: Microgenderome or microsexome? J Neurogastroenterol Motil. (2022) 28:332–3. doi: 10.5056/jnm21242 35362460 PMC8978118

[50] Cong X Xu W Janton S Henderson WA Matson A McGrath JM . Gut microbiome developmental patterns in early life of preterm infants: impacts of feeding and gender. PloS One. (2016) 11:e0152751. doi: 10.1371/journal.pone.0152751 27111847 PMC4844123

[51] Korpela K Kallio S Salonen A Hero M Kukkonen AK Miettinen PJ . Gut microbiota develop towards an adult profile in a sex-specific manner during puberty. Sci Rep. (2021) 11:23297. doi: 10.1038/s41598-021-02375-z 34857814 PMC8640005

[52] Markle JG Frank DN Mortin-Toth S Robertson CE Feazel LM Rolle-Kampczyk U . Sex differences in the gut microbiome drive hormone-dependent regulation of autoimmunity. Science. (2013) 339:1084–8. doi: 10.1126/science.1233521 23328391

[53] Shin JH Park YH Sim M Kim SA Joung H Shin DM . Serum level of sex steroid hormone is associated with diversity and profiles of human gut microbiome. Res Microbiol. (2019) 170:192–201. doi: 10.1016/j.resmic.2019.03.003 30940469

[54] Koren O Goodrich JK Cullender TC Spor A Laitinen K Backhed HK . Host remodeling of the gut microbiome and metabolic changes during pregnancy. Cell. (2012) 150:470–80. doi: 10.1016/j.cell.2012.07.008 22863002 PMC3505857

[55] Santos-Marcos JA Rangel-Zuniga OA Jimenez-Lucena R Quintana-Navarro GM Garcia-Carpintero S Malagon MM . Influence of gender and menopausal status on gut microbiota. Maturitas. (2018) 116:43–53. doi: 10.1016/j.maturitas.2018.07.008 30244778

[56] Kamada N Seo SU Chen GY Nunez G . Role of the gut microbiota in immunity and inflammatory disease. Nat Rev Immunol. (2013) 13:321–35. doi: 10.1038/nri3430 23618829

[57] Wang L Tang L Zhai D Song M Li W Xu S . The role of the sex hormone-gut microbiome axis in tumor immunotherapy. Gut Microbes. (2023) 15:2185035. doi: 10.1080/19490976.2023.2185035 36880651 PMC10012946

[58] Plottel CS Blaser MJ . Microbiome and Malignancy. Cell Host Microbe. (2011) 10:324–35. doi: 10.1016/j.chom.2011.10.003 22018233 PMC3264051

[59] Terekhova M Bohacova P Artyomov MN . Human immune aging. Immunity. (2025) 58:2646–69. doi: 10.1016/j.immuni.2025.10.009 41175875

[60] Paharkova-Vatchkova V Maldonado R Kovats S . Estrogen preferentially promotes the differentiation of CD11c+ CD11b(intermediate) dendritic cells from bone marrow precursors. J Immunol. (2004) 172:1426–36. doi: 10.4049/jimmunol.172.3.1426 14734718

[61] DeBolt CA Yu H Riis V Ncube L Horowitz A Elovitz MA . Revealing the complexity of immunobiological shifts from non-pregnant to pregnant state. Am J Reprod Immunol. (2025) 94:e70166. doi: 10.1111/aji.70166 40965855 PMC13045527

[62] Baines KJ West RC . Sex differences in innate and adaptive immunity impact fetal, placental, and maternal healthdagger. Biol Reprod. (2023) 109:256–70. doi: 10.1093/biolre/ioad072 37418168

[63] Yalcinkaya A Yalcinkaya R Sardh F Landegren N . Immune dynamics throughout life in relation to sex hormones and perspectives gained from gender-affirming hormone therapy. Front Immunol. (2024) 15:1501364. doi: 10.3389/fimmu.2024.1501364 39885993 PMC11779622

[64] St Clair LA Chaulagain S Klein SL Benn CS Flanagan KL . Sex-differential and non-specific effects of vaccines over the life course. Curr Top Microbiol Immunol. (2023) 441:225–51. doi: 10.1007/978-3-031-35139-6_9 37695431 PMC10917449

[65] Werner RJ Wirth JR Cunningham MA . Rebalancing immunity: The emerging role of selective estrogen receptor modulators in immunity and autoimmunity. Curr Trends Immunol. (2025) 25:79–98. 42232584 PMC13225871

[66] Vona R Cittadini C Ortona E Matarrese P . Sex disparity in cancer: Role of autophagy and estrogen receptors. Cells. (2025) 14. doi: 10.3390/cells14040273 39996745 PMC11854201

[67] Blessing AM Rajapakshe K Reddy Bollu L Shi Y White MA Pham AH . Transcriptional regulation of core autophagy and lysosomal genes by the androgen receptor promotes prostate cancer progression. Autophagy. (2017) 13:506–21. doi: 10.1080/15548627.2016.1268300 27977328 PMC5361609

[68] Kwon H Schafer JM Song NJ Kaneko S Li A Xiao T . Androgen conspires with the CD8(+) T cell exhaustion program and contributes to sex bias in cancer. Sci Immunol. (2022) 7:eabq2630. doi: 10.1126/sciimmunol.abq2630 35420889 PMC9374385

[69] Guan X Polesso F Wang C Sehrawat A Hawkins RM Murray SE . Androgen receptor activity in T cells limits checkpoint blockade efficacy. Nature. (2022) 606:791–6. doi: 10.1038/s41586-022-04522-6 35322234 PMC10294141

